# Modified look-locker inversion recovery T1 mapping indices: assessment of accuracy and reproducibility between MRI scanners

**DOI:** 10.1186/1532-429X-15-S1-P134

**Published:** 2013-01-30

**Authors:** Fabio S Raman, Nadine Kawel, Neville Gai, Melanie Freed, Jing Han, Peter Kellman, Chia-Ying Liu, Joao A Lima, David A Bluemke, Songtao Liu

**Affiliations:** 1 Radiology and Imaging Sciences, National Institutes of Health Clinical Center, Bethesda, MD, USA; 2 Facultad de Física - Pontificia Universidad Católica de Chile, Macul, Santiago, Chile; 3 U.S. Food and Drug Administration, Rockville, MD, USA; 4 Laboratory of Cardiac Energetics, National Heart, Lung and Blood Institute, Bethesda, MD, USA; 5 School of Medicine, Johns Hopkins University, Baltimore, MD, USA; 6 Molecular Biomedical Imaging Laboratory, National Institute of Biomedical Imaging and Bioengineering, Bethesda, MD, USA

## Background

Cardiac magnetic resonance (CMR) T1 mapping indices, including T1 time, partition coefficient (λ), and extracellular volume fraction (ECV) measurements, are a group of emerging noninvasive, quantitative imaging biomarkers that can be used to assess diffuse myocardial fibrosis. Similar to other quantitative MRI measurements, T1 mapping results can be influenced by multiple scanner dependent factors, such as field strength, gradient systems, coil configuration, pulse sequence design, and artifacts related to field inhomogeneity and eddy currents. The purpose of this ex-vivo phantom, multicenter study was to investigate how scanner and field strength variation affected the accuracy and precision of T1 mapping indices.

## Methods

MR studies were performed on two 1.5T (1 Philips, 1 Siemens) and three 3T (2 Philips, 1 Siemens) scanners. Two sets of four phantoms were made to mimic the T1/T2 of pre- and post-contrast myocardium and blood at 1.5T and 3T. T1 mapping using modified look locker with inversion recovery (MOLLI) was performed with simulated heart rate of 40-100bpm using a standard 17 heart beat and a shorter 11 heart beat MOLLI protocol. Inversion recovery spin echo (IR-SE) sequence with TR = 10 sec was acquired during the same session to estimate the reference T1. The phantoms were taken out the magnet after IR-SE and all MOLLI protocols were re-scanned after 10 min to evaluate inter-scan reproducibility. The partition coefficient (λ) was estimated by ΔR1myocardium/ΔR1blood. General linear model was used to compare the accuracy and reproducibility of T1 and λ across field strength, scanners and protocols.

## Results

The IR-SE T1 values were significantly different across scanners within the same field strength. The average partition coefficient estimated from IR-SE was 43.7% (1.5T) and 46.0% (3T), similar to normal in-vivo values. Accuracy was defined as the percent error between MOLLI and IR-SE, and scan/re-scan reproducibility was reported as the relative percent mean difference between the two back to back MOLLI scans. T1 and λ accuracy both varied significantly between different scanners (p<0.0001 for both T1 and λ) and across heart rates (p<0.0001 for T1 and p=0.001 for λ). However, neither varied across the standard 17HB and 11HB protocols (p=0.177 for T1 and p=0.574 for lamda). Additionally, field strength significantly affected T1 accuracy but not λ accuracy (p<0.0001 for T1 vs. p=0.109 for λ). In addition to less variability in accuracy, λ also had lower percent error overall between scan repetitions, or higher scan/re-scan reproducibility, than T1 measurements (4.59% vs. 5.54%).

## Conclusions

MOLLI T1 mapping indices, including both native T1 and λ, exhibited significant accuracy and precision variation across scanners. Compared with absolute native T1, relative T1 mapping indices, such as λ, has less variability in accuracy across platforms and field strength as well as higher scan/re-scan reproducibility, which is ideal for multicenter studies.

## Funding

Funded by the National Institutes of Health (NIH) Intramural program.

**Table 1:** T1 partition coefficient accuracy and reproducibility

a)	T1			Partition coefficient		
**1.5T scanners**	**Accuracy**	**Reproducibility**		**Accuracy**	**Reproducibility**	

**1**	6.70 ± 0.46	5.17 ± 0.44		7.93 ± 0.96	2.09 ± 0.92	

**2**	5.97 ± 0.41	5.45 ± 0.33		9.71 ± 0.85	10.67 ± 0.70	

**3T scanners**						

**3**	8.01 ± 0.41	4.71 ± 0.33		8.85 ± 0.85	6.36 ± 0.70	

**4**	13.91 ± 0.41	3.80 ± 0.33		8.60 ± 0.85	5.55 ± 0.70	

**5**	10.58 ± 0.41	3.07 ± 0.33		6.66 ± 0.85	2.21 ± 0.70	



**b)**	**Field strength**			**Protocol**		

	**1.5T**	**3T**	**p-value**	**17 HB**	**11 HB**	**p-value**

**T1 accuracy**	6.33 ± 0.31	10.83 ± 0.23	< 0.0001	8.34 ± 0.27	8.83 ± 0.27	0.177

**T1 reproducibility**	5.31 ± 0.27	3.86 ± 0.19	<0.0001	4.46 ± 0.23	4.71 ± 0.23	0.432

**λ accuracy**	8.82 ± 0.64	8.04 ± 0.49	0.109	8.32 ± 0.57	8.53 ± 0.57	0.574

**λ reproducibility**	6.38 ± 0.58	4.71 ± 0.40	0.021	4.50 ± 0.46	6.58 ± 0.46	0.003

Table 1a shows the accuracy and reproducibility results for the 5 scanners used in the multicenter study while b shows the dependence of field strength and protocol on accuracy and reproducibility. Data is presented as least square means ± standard error. Accuracy is reported as the percentage difference between MOLLI and IR-SE. Scan/rescan reproducibility is reported as the relative percent mean difference between two MOLLI scans. Scanners:1- Avanto, Siemens, 1.5T; 2- Achieva, Philips, 1.5T; 3- Verio, Siemens, 3T; 4- Achieva, Philips, 3T; 5- Achieva, Philips, 3T.

**Figure 1:**
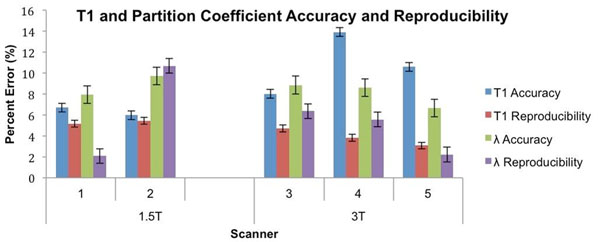
shows the accuracy and reproducibility measurements for both T1 and partition coefficient for each of the 5 scanners. Data is presented as least square means ± standard error. Accuracy is reported as the percentage difference between MOLLI and IR-SE. Scan/rescan reproducibility is reported as the relative percent mean difference between two MOLLI scans. Scanners:1- Avanto, Siemens, 1.5T; 2- Achieva, Philips, 1.5T; 3- Verio, Siemens, 3T; 4- Achieva, Philips, 3T; 5- Achieva, Philips, 3T.

